# Association of diet quality and morbidity profiles with health-seeking behavior among older adults in Noakhali, Bangladesh: A cross-sectional study

**DOI:** 10.1371/journal.pone.0330172

**Published:** 2025-11-14

**Authors:** Marjia Sultana, Md. Mehedi Hasan, Towhid Hasan, Israt Jahan Jui

**Affiliations:** Department of Food Technology and Nutrition Science, Noakhali Science and Technology University, Noakhali, Bangladesh; Niigata University, JAPAN

## Abstract

Diet quality and morbidity profiles significantly influence health outcomes among older adults. However, their association with health-seeking behavior remains understudied in Bangladesh. Hence, this study aimed to evaluate the association of diet quality and morbidity profiles with health-seeking behavior among older adults in Noakhali district, Bangladesh. This cross-sectional study was conducted from January to March 2024 among 400 adults aged ≥60 years at Noakhali General Hospital, Noakhali, Bangladesh. Data on socio-demographics, dietary patterns, morbidity profile, and health-seeking behavior were collected using a structured questionnaire. The mean Non-Communicable Disease (NCD)-Protect, NCD-Risk, and Global Dietary Recommendation (GDR) scores were 4.72, 1.94, and 11.77, respectively. Diabetes mellitus (98.2%) and musculoskeletal pain (44.3%) were the most prevalent morbidities. Around 30% of the participants visited healthcare providers at least once in a month. Consumption of pulses (adjusted odds ratio [aOR]: 1.927, p = 0.022), vitamin A-rich orange vegetables (aOR: 1.646, p = 0.040), and other fruits (aOR: 1.697, p = 0.034 was associated with higher healthcare utilization, while baked/grain-based sweets (aOR: 0.420, p = 0.015) and processed meat (aOR: 0.144, p < 0.001) were linked to lower healthcare use. Participants with musculoskeletal pain (aOR: 1.876, p = 0.013) and cardiovascular disease (aOR: 5.994, p = 0.003) were more likely to seek healthcare, while those with food allergies (aOR: 0.256, p < 0.001) and diabetes mellitus (aOR: 0.147, p = 0.047) were less likely. Moderate diet quality and specific morbidity profiles influence health-seeking behavior among older adults in Noakhali, Bangladesh. Therefore, the findings suggest that targeted dietary and healthcare interventions may enhance healthcare utilization and overall well-being of this vulnerable population.

## Introduction

A person passes a few stages in their total lifespan; old age is last to them. The range of ages for those who are getting close to or beyond life expectancy is known as old age (≥ 60 years), generally characterized by wisdom [1] and unique challenges [2]. Worldwide, the average lifespan of the population is rising rapidly [3]. Consequently, there will be 2.1 billion people aged over 60 years by 2030 [4], and the proportion of people over 60 years may increase from 12% (2015) to 22% (2050) [3]. Bangladesh has also observed an unparalleled rise in the aging population rate in recent decades, possibly due to an increase in life expectancy and a decrease in birth rate [5].

Diet quality is central to maintaining health, especially in older adults [6]. Diet quality refers to the overall healthfulness of a dietary pattern, characterized by the variety of foods consumed and their alignment with dietary guidelines [7]. Most Bangladeshi people, including elderly individuals, depend on rice as a staple food, making it more challenging to maintain a balanced diet [8]. In older age, the dietary habit gradually shifted from higher intake of vegetables, legumes, fruits, nuts, either whole grains, cereals, or non-refined grains, fish, and unsaturated vegetable oils to a higher consumption of animal products, sugary foods and fats and oils [9]. Consequently, poor diet quality causes micronutrient deficiencies and other diet-related non-communicable illnesses among them. Besides, many elder people may have limited access to nutritious food due to poverty, physical limitations, or lack of family support, which can exacerbate their health problems [10].

Older people are more susceptible to illnesses and injuries than younger adults due to physiological and social changes, such as reduced food intake, diminished sensory perception, malabsorption, compromised immunity, reduced healing abilities, ageism, loneliness, and retirement-related cultural issues [11,12]. The presence of individual or combinations of multiple acute and chronic conditions often characterizes the morbidity status of older adults. In Bangladesh, the common health issues faced by older adults include diabetes mellitus, cardiovascular disease, food allergies, hypertension, hearing problems, visual impairment, dental problems, musculoskeletal pain, bedsores, sense of thirst, respiratory disorders, etc. These health problems impact their physical well-being and mental and social health, leading to a diminished quality of life [13].

Health-seeking behavior is another critical factor that has an influence on the health outcomes of individuals. It refers to the decisions and actions individuals take to maintain their health and seek appropriate care when needed [14]. Various factors influence health-seeking behavior, including accessibility to healthcare services, affordability, cultural beliefs, and knowledge of health conditions [15]. In Bangladesh, older people may face barriers such as a lack of nearby healthcare facilities, high out-of-pocket costs, and social stigmas around seeking care, especially in rural areas. Additionally, older adults sometimes feel reluctant to seek necessary medical intervention while needed due to perceived age-related decline or a dependency on traditional remedies, leading to worsened health outcomes [16].

South Asia, including Bangladesh, has witnessed notable changes in dietary patterns along with health and morbidity status, especially among elderly, due to the rapid urbanization, globalization, and socio-economic transitions. Hence, understanding the patterns of health-seeking behavior and their association with dietary habits and morbidity profiles is vital to enhancing the well-being and quality of life of older adults in Bangladesh, ensuring they receive the care and support they need to lead healthier aging [16–19]. Investigating these issues will make it possible to close the present gaps in healthcare policies and delivery to minimize the prevalence of malnutrition and chronic diseases among older people [20]. However, to the best of our knowledge, no studies have been conducted yet to investigate the association of diet quality and morbidity profile with health‑seeking behavior among older people in Bangladesh. Thus, this study aims to evaluate the association of diet quality and morbidity profile with health‑seeking behavior among older people in Noakhali district, Bangladesh. This study hypothesizes that higher diet quality, characterized by greater adherence to dietary recommendations, is positively associated with increased health-seeking behavior among older adults. Additionally, specific morbidity profiles may significantly influence the frequency of healthcare utilization.

## Methods

### Study design, setting and participants

This cross-sectional study was conducted among older adults seeking care at the outpatient department (OPD) of Noakhali General Hospital, Noakhali, Bangladesh. Noakhali General Hospital is a primary referral center for the entire district, attracting patients from both urban and rural areas. Its strategic location and reputation for providing essential healthcare services make it a destination for individuals from diverse socio-economic and demographic backgrounds of Noakhali. This single-center setting ensures that the participants of this study indirectly represent/generalize the broader population of Noakhali by capturing healthcare-seeking patterns across various unions and upazilas.

The study duration was from 29/01/2024–03/03/2024. The elderly individual was defined as a person aged ≥ 60 years, according to the National Policy on Older Persons 2013 in Bangladesh [21]. The inclusion criteria were considered as follows: (1) living in Noakhali district for at least one year, and (2) aged ≥ 60 years. Those elderly who were critically ill (e.g., those requiring emergency care, bedridden, or with known cognitive impairments or memory-related disorders) and individuals unwilling/unable to provide informed consent were excluded from the study. The sample size of the study was calculated using Cochran’s formula of z^2^ p (1-p)/ d^2^ [22]. Here, z was the value of the normal distribution (at a 95% confidence level, it was 1.96); p was the expected prevalence, which was assumed to be 50% in this case; d value around 0.05 was the tolerable standard error. This expected prevalence was chosen as a conservative estimate in the absence of prior data on the prevalence of health-seeking behavior among older adults in Noakhali, Bangladesh. Using p as 50% ensures the maximum sample size, providing sufficient statistical power to detect significant associations within the study population.

According to this equation, the total sample size was 384. To account for potential attrition or non-response, the calculated sample size was increased by 4%, resulting in a final target of 400 participants. This adjustment ensured the study maintained adequate power despite incomplete responses or participant withdrawals. The sampling frame consisted of elderly patients (aged ≥60 years) listed in the daily OPD registry of Noakhali General Hospital. A simple random sampling technique was applied using a computer-generated random number table to select participants. A total of 405 older adults visiting the OPD during the study period were approached to ensure unbiased representation and finally data were collected from 400 individuals (response rate > 98%). This method minimized selection bias and enhanced the generalizability of the findings to the broader elderly population in Noakhali.

### Questionnaire development

A structured questionnaire was prepared based on previous literature [13,16,23,24]. Two public health experts reviewed the draft. Further, the questionnaire was modified based on their suggestions and comments. A pilot study was also carried out from 10/01/2024–15/01/2024 with twenty-five participants (around 5% of the total sample size) to assess the face validity and reliability of the questionnaire. Finally, a pretested and standardized questionnaire was prepared for data collection (S1 File).

To address potential recall bias, the questionnaire was designed with straightforward and easily understandable questions to facilitate accurate recall. Additionally, participants were asked to recall dietary intake from the previous day (24-hour dietary recall) to minimize memory-related inaccuracies. Furthermore, the inclusion and exclusion criteria were applied as mentioned above to ensure the reliability and validity of the collected data.

#### Socio-demographic and anthropometric information.

The socio-economic and demographic characteristics of the participants, such as gender, age, marital status, family size, occupation, education, household income, etc., were collected. The anthropometric measurements included weight and height. Weight was measured using a digital weighing scale (SECA 813, Hamburg, Germany) to the nearest 0.1 kg, and height was measured using a portable stadiometer (SECA 213, Hamburg, Germany) to the nearest 0.1 cm. The nutritional status of the participants was categorized according to the World Health Organization’s Asian BMI cut-offs, where underweight, normal, overweight, pre-obese, and obese categories were defined when BMI was < 18.5, 18.5–22.9, 23–24.9, 25–29.9 and ≥ 30 kg/m2, respectively [25].

#### Nutrition related characteristics.

The simplified nutrition appetite questionnaire (SNAQ) was used to measure the participants’ nutrition-related attributes. SNAQ is a validated screening tool for nutritional assessment [13]. The questions were divided into four areas: appetite, satiety, taste of food, and number of meals a person usually has daily. The total score was 4–20 points, and a cutoff level of ≤14 was considered as a risk of malnutrition [26].

The mini nutritional assessment (MNA) is another valid scale for screening and assessing malnutrition among the elderly population worldwide [27] and the Mini Nutritional Assessment – Short Form (MNA-SF) was employed in this study. This questionnaire contained six items: decreased food intake, weight loss during the last three months, mobility, recent psychological stress or acute disease, neuropsychological problems, and the Body Mass Index (BMI). The maximum score was 14, and 0–7, 8–11, and 12–14 were considered malnourished, at risk of malnutrition, and normal nutritional status, respectively [28].

#### Dietary behavior.

Diet quality was assessed by the diet quality questionnaire (DQQ) which was modified so as to represent the foods accessible in Bangladesh. DQQ is a nutrition assessment tool with a quick procedure, which helps with accurate measurement and tracking of diet quality at population level [29]. Since the objective of this questionnaire is to collect data on the food group consumption rate for determining diet quality indices among the global public, it has been implanted/validated in 55 countries in 2021–2022 of the Gallup World Poll [6,29,30] along with in Bangladesh among adolescent [31]. Consequently, its adaptation in this study followed standard procedures to reflect the dietary patterns specific to older adults in Bangladesh. The DQQ for Bangladesh had the food items list of 29 food groups that are necessary for calculating minimum dietary diversity along with the additional food groups required for indicators associated with risk factors of chronic diseases. The participants were instructed to have a recall of the foods that were consumed by them throughout the previous day.

Three scores named “NCD-Protect,” “NCD-Risk,” and “Global Dietary Recommendation (GDR) score,” was calculated from the DQQ data by which diet quality of the participants could be feasibly monitored. Among these, NCD-Protect included nine categories of foods: 1) whole grains; 2) pulses; 3) nuts and seeds; 4) vitamin A–rich orange vegetables; 5) dark green leafy vegetables; 6) other vegetables; 7) vitamin A–rich fruits; 8) citrus; and 9) other fruits. However, NCD-Risk considered eight categories of foods: 1) soft drinks (sodas); 2) baked/grain-based sweets; 3) other sweets; 4) processed meat (double weighted); 5) unprocessed red meat; 6) deep fried food; 7) fast food and instant noodles; and 8) packaged ultra-processed salty snacks. The GDR score reflects all eleven recommendations which was further measured by deducting the NCD-Risk scores from the NCD-Protect scores. The range of both NCD-Protect and NCD-Risk scores was from 0 to 9 where the GDR score ranged from −9–9. Lower NCD-Protect and GDR but higher NCD-Risk score reflects poor diet quality [32].

#### Morbidity characteristics.

The incidence of diseases of the participants was recorded by asking them using structured close ended questions developed based on standardized health assessment tools, for example, “Do you suffer from any of these health problems (e.g., diabetes mellitus, cardiovascular disease, food allergies, hypertension, hearing problem, visual impairment, dental problems, musculoskeletal pain, bedsore, sense of thirst etc.)?” Participants had two option “Yes” or “No. If they had any other disease that had not asked in the questionnaire were also recorded. Additionally, available medical records, such as diagnostic reports and physician notes, were reviewed to validate the self-reported data whenever possible, reducing the risk of bias and inaccuracies. The choice of this dual approach (self-reports complemented by the review of available medical records) was made to ensure the inclusion of all relevant morbidity data while maintaining the study’s feasibility within the given timeframe and resources.

#### Health-seeking behavior.

Participants were asked about the frequency of visits to a healthcare provider or seeking medical care. The responses were categorized into five groups; several times per month, once per month, several times per year, once per year and less regular than once per year. Participants were considered to have appropriate health-seeking behavior if they sought health cate at least once per month.

### Data collection

Data collectors were final-year undergraduate students from the Department of Food Technology and Nutrition Science at Noakhali Science and Technology University. They underwent a comprehensive training program which covered essential topics such as the study’s objectives, data collection procedures, and ethical considerations. The training also included role-playing exercises and mock interviews to enhance their interviewing skills and ensure consistency in data collection. Additionally, the data collectors were supervised by experienced researchers throughout the data collection process to address any challenges in real time.

With the help of the data collectors, the authors approached the elderly seeking care at Noakhali General Hospital. The objectives of the study, anticipated benefits, and potential risks of participating were clearly explained to them to encourage active participation and cooperation. They were also assured of anonymity and confidentiality and allowed to skip interviews at any study stage. Finally, information was gathered from individuals who consented to join the study through face-to-face interviews, which took approximately 20 minutes to complete.

### Ethical consideration

The study was approved by the Ethical Committee of Noakhali Science and Technology University (reference number: NSTU/SCI/EC/2023/199). Every experimental process was fitted with the relevant standards and regulations. Both oral and written informed consent was obtained from the participants before data collection.

### Statistical analysis

The collected data were analyzed using Statistical Package for the Social Sciences (SPSS, vs. 27) for Windows (SPSS Inc., Chicago, IL, USA). For the continuous variables, mean and standard deviation (SD), and for categorical variables, frequency and percentage were employed where applicable. A multiple logistic regression was used to determine the association of diet quality and morbidity status with appropriate health-seeking behavior among the participants. A p-value of < 0.05 (two-tailed) was defined as statistically significant for all tests.

## Results

Table 1 shows the participants characteristics. This study included 198 male and 202 female elderly participants. A majority of the participants were aged 60–69 years and the population predominantly lived in rural settings. About one in ten participants had no formal education. The occupational status shows that around 40% had no cash income at their current age. In terms of marital status, about three-fourths were currently married and approximately one-fourth of the participants had normal nutritional status. Almost equal proportion of the participants were at risk of being malnourished and well-nourished, as revealed by their SNAQ scores. Besides, according to the MNA scores, more than two-fourths were at risk of malnutrition. The majority of the participants had an income ≥ 30,000 BDT and had 5–8 members in their family. The mean (SD) physical activity scale for the elderly (PASE) score of the elderly was 86.67 (49.9).

**Table 1:** Participants characteristics (n = 400).

Variables	Category	Frequency	%
Gender	Male	198	49.5
	Female	202	50.5
Age (years)	60-69	330	82.5
	70-79	62	15.5
	≥80	8	2.0
Residence	Rural	225	56.3
	Urban	175	43.7
Level of education	No formal schooling	43	10.8
	Primary	145	36.2
	Secondary	152	38.0
	Tertiary	60	15.0
Occupation	Service	56	14.0
	Self-employed	181	45.3
	No cash income	163	40.7
Marital status	Never married	2	0.5
	Currently married	300	75.0
	Divorced/separated/widowed	98	24.5
BMI (kg/m^2^)	<18.5	5	1.3
	18.5–22.9	106	26.4
	23.0–24.9	149	37.2
	25.0–29.9	125	31.3
	≥30	15	3.8
SNAQ score	≤14	195	48.8
	>15	205	51.2
MNA score	Malnourished	24	6.0
	At risk of malnutrition	182	45.5
	Normal nutritional status	194	48.5
Monthly household income (BDT)	≤30000	102	25.5
	>30000	298	74.5
Household member	1-4	132	33.0
	5-8	261	65.2
	>8	7	1.8

BDT: Bangladeshi taka, BMI: Body mass index, MNA: Mini nutritional assessment, SNAQ: Simplified nutritional appetite questionnaire.

The proportion of the study population consuming various food groups is detailed in Table 2. Almost all the participants consumed whole grains, with a higher percentage consumed pulse. Intake of vitamin A-rich vegetables and fruits and citrus fruits was less prevalent, where the majority of the participants consumed dark green leafy vegetables, other vegetables, and other fruits. Around 40% of the participants consumed unhealthy foods like deep-fried foods, fast foods, and instant noodles. However, the intake of nuts and seeds, soft drinks, other sweets, processed meat, and unprocessed red meat was much lower (less than 15%). The mean NCD-Protect score among the participants was 4.72, indicating moderate adherence to a diet protecting against NCDs. Conversely, the average NCD-Risk score was 1.94, which reflects lower consumption of high-risk foods. Additionally, the GDR score that balances protective and risk factors in the diet was observed to be around 11.00 among the participants, indicating overall moderate diet quality.

**Table 2:** Diet quality of the participants (n = 400).

Variables	No (%)	Yes (%)
Whole grains	4 (1.0)	396 (99.0)
Pulses	110 (27.5)	290 (72.5)
Nuts and seeds	350 (87.5)	50 (12.5)
Vitamin A-rich orange vegetables	254 (63.5)	146 (36.5)
Dark green leafy vegetables	137 (34.3)	263 (65.7)
Other vegetables	51 (12.8)	349 (87.2)
Vitamin A-rich fruits	340 (85.0)	60 (15.0)
Citrus	312 (78.0)	88 (22.0)
Other fruits	156 (39.0)	244 (61.0)
Soft drinks	399 (99.7)	1 (0.3)
Baked/grain-based sweets	322 (80.5)	78 (19.5)
Other sweets	364 (91.0)	36 (9.0)
Processed meat	340 (85.0)	60 (15.0)
Unprocessed red meat	340 (85.0)	60 (15.0)
Deep fried food	223 (55.8)	177 (44.2)
Fast food & Instant noodles	219 (54.7)	181 (45.3)
Packaged ultra-processed salty snacks	276 (69.0)	124 (31.0)
NCD-Protect score (mean±SD)	4.72 ± 1.16	
NCD-Risk score (mean±SD)	1.94 ± 1.42	
GDR score (mean±SD)	11.77 ± 1.65	

GDR: Global Dietary Recommendations, NCD: Non-communicable disease.

**Fig 1:**
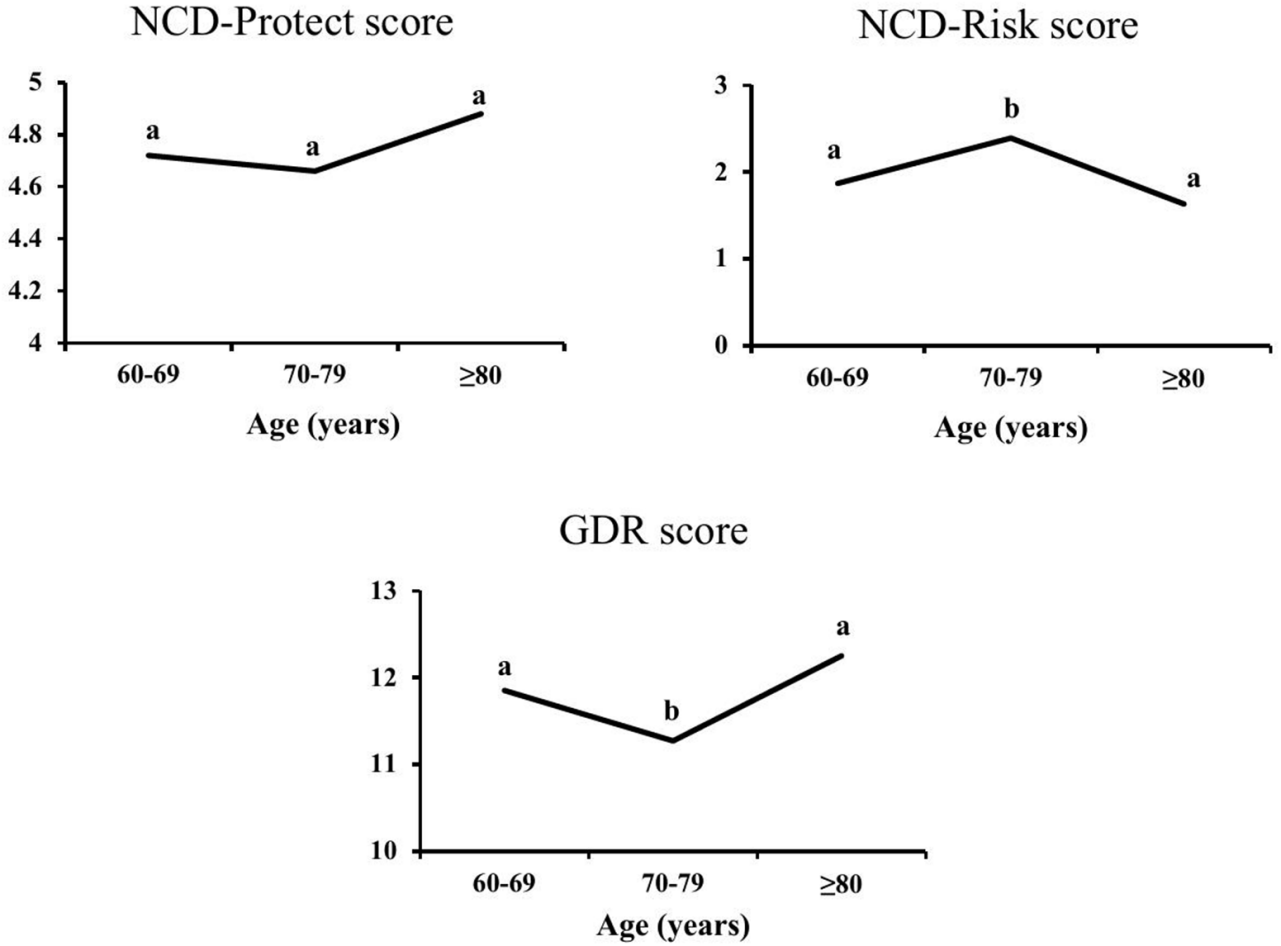
Diet quality scores of the participants in different age category. a–bValues with the different superscript letter within the same category are significantly (p < 0.05) different.

Fig 1 delineates the participants’ diet quality by their NCD-Protect, NCD-Risk, and GDR scores, categorized by age groups of 60–69 years, 70–79 years, and ≥ 80 years. NCD-Protect score reflecting the consumption of fruits, vegetables, whole grains, legumes, and nuts that protect against NCDs, shows a U-shaped pattern where the oldest age group (≥ 80 years) had the highest scores (4.88), with the lowest score among the group of 70–79 years (4.66). The NCD-Risk score, which indicates the intake of foods that enhance NCD risk, including meats, processed foods, sweetened foods, and foods rich in saturated fats, shows an inverse pattern compared to the NCD-Protect score. The score was highest among the group of 70–79 years, suggesting that individuals in this age range consume more of these risk-associated foods, with the lowest score in the ≥ 80 age group. Lastly, the GDR score, a comprehensive measure that balances the protective and risk factors in the diet, mirrors a U-shaped pattern similar to the NCD-Protect score. The GDR score peaked in the ≥ 80 years group but was lowest among the group of 70–79 years. Overall, the patterns of Fig 1 suggested that diet quality was somewhat lower in the 70–79 age group, with higher consumption of risk-related foods and lower intake of protective foods. In contrast, the diet quality improves slightly in those aged 80 years and older.

Table 3 represents the prevalence of various morbidity conditions among the participants. Among the health conditions listed in Table 3, diabetes mellitus and musculoskeletal pain were the most common morbidities, while cardiovascular disease and bedsores were less prevalent.

**Table 3:** Morbidity status of the participants (n = 400).

Variables	Absent (%)	Present (%)
Visual impairment	244 (61.0)	156 (39.0)
Hearing problem	353 (88.2)	47 (11.8)
Musculoskeletal pain	223 (55.7)	177 (44.3)
Bedsore	398 (99.5)	2 (0.5)
Food allergy	286 (71.5)	114 (28.5)
Sense of thirst	374 (93.5)	26 (6.5)
Dental problem	293 (73.3)	107 (26.7)
Diabetes mellitus	7 (1.8)	393 (98.2)
Cardiovascular disease	382 (95.5)	18 (4.5)
Hypertension	292 (73.0)	108 (27.0)

**Fig 2:**
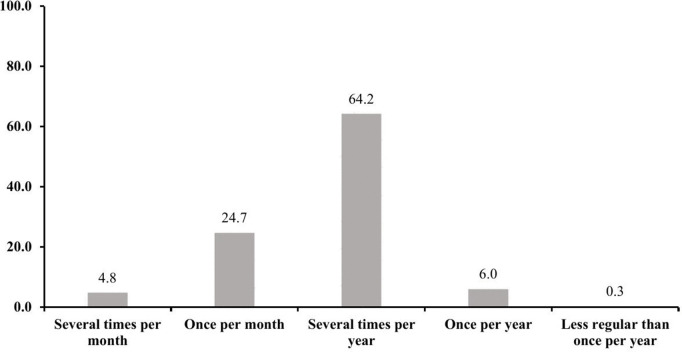
Frequency of health seeking behavior among the participants.

Fig 2 illustrates the frequency of health-seeking behavior among the participants into five categories: several times per month, once per month, several times per year, once per year, and less regularly than once per year. Most participants visited the healthcare providers several times per year, followed by 24.7% who visited healthcare once per month. A smaller proportion of participants seek healthcare once per year, several times per month, and less than once per year.

Table 4 presents the adjusted odds ratios (aOR) with 95% confidence intervals (CI) for various food groups and their correlation with health-seeking behavior among older people. The study participants who consumed pulses, vitamin A-rich orange vegetables, and other fruits had a significantly higher likelihood of engaging in appropriate health-seeking behavior, suggesting that these dietary components enhanced their health-seeking actions. Besides, the intake of baked/grain-based sweets and processed meat significantly reduced appropriate health-seeking behavior, with an aOR of 0.420 (p = 0.015) and 0.144 (p < 0.001), respectively.

**Table 4:** Association of diet quality with appropriate health-seeking behavior among the participants (n = 400).

Variables	aOR (95% CI)	P-value
*Whole grains*		
No	1	
Yes	0.245 (0.032–1.862)	0.174
*Pulses*		
No	1	
Yes	1.927 (1.101–3.372)	0.022
*Nuts and seeds*		
No	1	
Yes	1.673 (0.876–3.195)	0.119
*Vitamin A-rich orange vegetables*		
No	1	
Yes	1.646 (1.023–2.647)	0.040
*Dark green leafy vegetables*		
No	1	
Yes	1.486 (0.910–2.427)	0.113
*Other vegetables*		
No	1	
Yes	1.777 (0.820–3.854)	0.145
*Vitamin A-rich fruits*		
No	1	
Yes	1.055 (0.533–2.086)	0.879
*Citrus*		
No	1	
Yes	1.074 (0.613–1.884)	0.802
*Other fruits*		
No	1	
Yes	1.697 (1.041–2.767)	0.034
*Soft drinks*		
No	1	
Yes	–	–
*Baked/grain-based sweets*		
No	1	
Yes	0.420 (0.209–0.847)	0.015
*Other sweets*		
No	1	
Yes	1.668 (0.791–3.518)	0.179
*Processed meat*		
No	1	
Yes	0.144 (0.049–0.423)	<0.001
*Unprocessed red meat*		
No	1	
Yes	1.023 (0.543–1.930)	0.943
*Deep fried food*		
No	1	
Yes	0.989 (0.626–1.563)	0.962
*Fast food & Instant noodles*		
No	1	
Yes	0.663 (0.413–1.063)	0.088
*Packaged ultra-processed salty snacks*		
No	1	
Yes	1.023 (0.622–1.680)	0.930

aOR: Adjusted odds ratio, CI: Confidence interval. All regression models were adjusted for gender, age (years), residence, level of education, occupation, marital status, BMI (kg/m^2^), SNAQ score, MNA score, monthly household income (BDT), household member, and PASE score.

The association between various morbidity conditions and appropriate health-seeking behavior among the elderly participants is illustrated in Table 5. The table reveals that participants suffering from musculoskeletal pain and cardiovascular disease were significantly more likely to engage in appropriate health-seeking behavior, with aOR of 1.876 (p = 0.013) and 5.994 (p = 0.003), respectively, than the participants who did not suffer. Conversely, the elderly subjects having food allergies (aOR: 0.256; p < 0.001) and diabetes mellitus (aOR: 0.256; p < 0.001) were less likely to seek appropriate healthcare compared to those who did not have these morbidity conditions.

**Table 5:** Association of morbidity status with appropriate health-seeking behavior among the participants (n = 400).

Variables	aOR (95% CI)	P-value
*Visual impairment*		
Absent	1	
Present	0.613 (0.363–1.036)	0.068
*Hearing problem*		
Absent	1	
Present	0.875 (0.401–1.907)	0.736
*Musculoskeletal pain*		
Absent	1	
Present	1.876 (1.140–3.086)	0.013
*Bedsore*		
Absent	1	
Present	–	–
*Food allergy*		
Absent	1	
Present	0.256 (0.132–0.496)	<0.001
*Sense of thirst*		
Absent	1	
Present	1.035 (0.384–2.787)	0.946
*Dental problem*		
Absent	1	
Present	1.689 (0.997–2.862)	0.051
*Diabetes mellitus*		
Absent	1	
Present	0.147 (0.022–0.977)	0.047
*Cardiovascular disease*		
Absent	1	
Present	5.994 (1.868–19.229)	0.003
*Hypertension*		
Absent	1	
Present	0.920 (0.507–1.669)	0.783

aOR: Adjusted odds ratio, CI: Confidence interval. All regression models were adjusted for gender, age (years), residence, level of education, occupation, marital status, BMI (kg/m^2^), SNAQ score, MNA score, monthly household income (BDT), household member, and PASE score.

## Discussion

Most participants were aged between 60–69 years, which reflects the overall demographic trend in Bangladesh, where improvements in healthcare have led to increased life expectancy [33]. Still, the proportion of the very old (≥ 80 years) remains relatively small. Lower educational attainment among the elderly participants observed in Table 1 is consistent with the general educational trends among older people in Bangladesh [34], where access to education was historically limited, especially for women and the rural population. About 40.7% of participants having no cash income mirrors the financial vulnerability of elderly individuals in Bangladesh. Besides, almost 50% of the participants were self-employed, suggesting they may engage in informal labor or subsistence farming activities. The high percentage of the married and low percentage of divorced/ not married individuals observed in this study are also typical of South Asian societies, where marriage rates are high, and divorce rates are low [35].

Only 26.4% of the participants had a normal nutritional status measured by their BMI, with a significant proportion being overweight (37.2%) or pre-obese (31.3%). This finding indicates the nutrition transition happening in many developing countries like Bangladesh, where traditional diets are replaced by more calorie-dense, processed foods, increasing overweight and obesity, even among the older population [10]. Besides, the prevalence of undernutrition among older people may be due to poverty and food insecurity [36]. A higher prevalence of normal nutritional status was observed when using the SNAQ or MNA scale compared to BMI. This can be attributed to the different aspects of nutritional status that each method assesses. For instance, BMI is a straightforward measure based solely on weight and height and does not consider muscle mass, fat distribution, or overall health [37]. As a result, it may categorize an elderly individual with low muscle mass as malnourished, even if their overall health and nutritional intake are adequate (having normal nutritional status) [38]. Conversely, the SNAQ and MNA are comprehensive tools that consider not just body measurements but also other factors such as dietary intake, recent weight loss, physical activity, mobility, appetite, and psychological health to more accurately identify the individuals who are not genuinely malnourished but might have a low BMI for other reasons [39].

The overall diet quality of the elderly participants of this study, mainly those aged 70–79 years, measured by the NCD-Protect, NCD-Risk, and GDR score, indicates a moderate adherence to healthy diets that included low intake of vitamin A-rich fruits and vegetables but high consumption of unhealthy foods such as deep-fried foods, fast foods, and instant noodles. The findings suggested that although the participants have some protective dietary habits, the presence of risk factors remains significant. Thus, this duality might cause the “double burden” of malnutrition, including under- and over-nutritional status, which is observed in this study and measured by their BMI. The findings of this study are in line with the previous studies, which reported a dietary transition towards higher consumption of processed and calory-dense foods, leading to the increased prevalence of over-nutritional status and NCDs among older people [10,40]. Mridha et al. [40] found a significant proportion of older people in Bangladesh consume an inadequately diversified diet. In addition, Hasan et al. [41] highlighted the double burden of malnutrition, with both underweight and obesity prevalent among older adults in Bangladesh, reflecting the nutritional transition mentioned earlier. These findings emphasize the urgent need for targeted interventions to reduce the intake of unhealthy foods and increase the consumption of healthy, protective foods to minimize the risk of over-nutritional status and NCDs.

Comorbidity existed among the elderly participants of this study, where diabetes mellitus and musculoskeletal pain were highly prevalent. Razon et al. [13] also reported 56.6% and 39.4% prevalence of diabetes and musculoskeletal pain among older people living in rural areas of Bangladesh, respectively. In addition, a significant burden of NCDs, such as diabetes, coronary artery disease, and ocular problems, etc. among the elderly was reported in India [42,43], mirroring the findings of the present study. However, the higher occurrences of NCDs in this study could be linked to their poor to moderate diet quality observed by their mean NCD-Protect, NCD-Risk score, and GDR score (Table 2). Interestingly, cardiovascular disease and bedsores were less prevalent than expected, which may reflect better mobility or care practices among the participants [44]. However, this finding contrasts with the previous studies because cardiovascular issues are typically more prevalent among older adults globally. For example, in Bangladesh, a high prevalence of comorbidity among the rural elderly was observed by Khanam et al. [45] with CVDs being among the most common conditions. These dissimilarities could be due to the differences in lifestyle patterns, availability and accessibility of healthcare facilities in different areas, or diagnostic practices in the studied population [46–48].

The majority of the participants of this study did not engage in frequent health-seeking behavior; instead, they depended on periodic check-ups or visits (Fig 2). Table 4 shows that consuming pulses, vitamin A-rich orange vegetables, and other fruits significantly increased health-seeking behavior among older adults, while intake of baked/grain-based sweets and processed meat reduced it. The findings of this study align with previous studies conducted in Bangladesh [40] and other countries [49,50], demonstrating the critical role of diet quality in influencing health-seeking behavior among older adults. Inadequate dietary diversity among older adults in Bangladesh has been observed to be linked with poor health outcomes and less health care utilization [40]. The present study underscores the importance of maintaining a nutritious diet to promote proactive health-seeking behavior among older adults across different cultural and socioeconomic contexts.

In Table 5, it was observed that the participants with food allergies and diabetes mellitus had less tendency to engage in health-seeking behavior, maybe because these conditions do not always cause immediate discomfort; thus, consistent healthcare utilization is not necessary [51]. Additionally, reasons such as cost, perception of illness, or healthcare barriers might prevent them from getting proper care for such conditions. The pattern of infrequent health-seeking behavior of the elderly participants observed in Fig 2 aligns with the previous studies [52–54] which also highlighted their visit to the healthcare providers only when sick/symptoms become severe. The irregular health-seeking behavior of the elderly individual in this study could be a reflection of a few factors, such as lack of health awareness, limited availability and accessibility to healthcare facilities, financial and physical constraints, lack of transportation, and cultural attitudes toward healthcare and others [53,55,56].

In this study, the participants who routinely sought health care services (Fig 2) may be for their chronic health conditions, as few of them reported having musculoskeletal pain, hypertension, cardiovascular disease, visual impairment, or dental problems (Table 2) that may push them to the more frequent visit of healthcare facilities [14,16,57]. The association between morbidity conditions and health-seeking behavior from Table 5 also confirmed that the elderly individuals who had musculoskeletal pain and cardiovascular disease were significantly more likely to seek healthcare services. This could be due to the fact that morbidity chronic conditions causing significant discomfort or posing severe health risks might have motivated the elderly people to seek health care more consistently, while other conditions might be associated with less frequent healthcare utilization [14,16,57]. These findings also align with the earlier studies that highlighted that chronic morbidity conditions might cause serious health risks that often motivate regular healthcare utilization [57,58]. For instance, the older adults of northern Kerala suffering from NCDs like cardiovascular disease sought regular health care monitoring because of the necessity of ongoing health management [57].

The present study underscored the importance of diet quality in influencing health-seeking behavior, where healthier diets rich in pulses, vitamin A-rich orange vegetables, and other fruits promoted more frequent and proactive healthcare utilization, and the diet containing baked/grain-based sweets and processed meat significantly reduced it. The findings suggested the positive effects of a healthy diet and the adverse effects of unhealthy foods on health consciousness, likely due to the connection between healthier diets and better health outcomes, leading to increased health awareness. Conversely, unhealthy diets in processed foods cause poorer health and well-being, reducing the motivation or ability to seek health care services regularly [59,60]. Thus, the findings of this study may have practical implications for healthcare practitioners working with elderly populations in Bangladesh and similar contexts. Based on the observed dietary patterns and associated health-seeking behaviors, several targeted recommendations can be made. Healthcare providers should implement community-based nutrition programs promoting nutrient-rich foods like pulses, vitamin A-rich vegetables, and fruits, targeting older adults in both urban and rural areas. Policymakers should integrate routine dietary assessments and counseling into healthcare services while ensuring access to affordable supplements for malnutrition-prone individuals. Health promotion campaigns must emphasize regular check-ups, particularly for those with chronic conditions. Collaboration among healthcare institutions, community organizations, and policymakers can enhance these efforts, ultimately improving health outcomes for the aging population. Future longitudinal studies should be conducted to track the dietary changes over time among the older adults to determine their long-term impacts on morbidity and healthcare-seeking behavior. This may provide a clearer understanding of causal relationships and the effectiveness of interventions designed to improve diet quality and health outcomes in this population.

### Limitations

The study has several limitations. First, the cross-sectional design limits the ability to establish causality between diet quality, morbidity profiles, and health-seeking behavior. Longitudinal studies are needed to assess changes over time. Second, the reliance on self-reported dietary and morbidity data introduces potential recall bias, as participants may not accurately remember past dietary intake or health-related information. Despite efforts to minimize this bias through the use of a 24-hour dietary recall and cognitive screening, some inaccuracies may persist. Additionally, the study was conducted in a single district, limiting the generalizability of the results to other regions of Bangladesh. Lastly, using BMI as a primary indicator of nutritional status does not account for muscle mass or fat distribution, potentially misclassifying some participants’ health conditions. Future studies should incorporate more comprehensive measures of health and nutrition to address these limitations. Future analyses may also explore the relationship between anthropometric and appetite-based measures (i.e., BMI, SNAQ, and MNA scores) through cross-tabulations or interaction models to further elucidate their combined impact on health-seeking behavior.

## Conclusion

The present study highlighted the critical association between diet quality, morbidity profiles, and health-seeking behavior among older adults in Noakhali district, Bangladesh. The findings revealed that moderate diet quality, characterized by high consumption of unhealthy foods and inadequate intake of protective foods, is common among older people. Diabetes mellitus and musculoskeletal pain were more prevalent among the participants. Importantly, healthier diets, rich in pulses, vitamin A-rich vegetables, and fruits, were associated with more frequent and proactive healthcare-seeking behavior. In contrast, diets with high processed food intake led to reduced health-seeking behavior, suggesting that improving diet quality and healthcare access is vital to enhancing the health outcomes and quality of life of older adults in Bangladesh. Older adults with musculoskeletal pain and cardiovascular disease were notably more likely to involve in appropriate healthcare-seeking behavior. In contrast, those with food allergies and diabetes mellitus were less likely to seek necessary medical care compared to individuals without these conditions.

Overall, the findings of this study highlight the significant role of diet quality and morbidity profiles in shaping health-seeking behavior among older adults in Noakhali, Bangladesh. To improve these outcomes, policymakers should implement community-based nutrition education programs, integrate dietary assessments into routine healthcare services, and establish mobile healthcare units to enhance access in rural areas. Public health initiatives should focus on elderly nutrition counseling, promoting healthier dietary patterns and regular health check-ups, particularly for those with chronic conditions. Collaborative efforts between healthcare providers, community organizations, and government agencies are essential to support healthy aging and improve overall health outcomes for the elderly population.

## Supporting information

S1 File Questionnaire. (PDF)
