# Supplementary material for: Association of diet quality and morbidity profiles with health-seeking behavior among older adults in Noakhali, Bangladesh: A cross-sectional study
Source: PLoS One. 2025 Nov 14;20(11):e0330172. doi: 10.1371/journal.pone.0330172 (PMC12617947; doi:10.1371/journal.pone.0330172)
Supplement: S1 File — (PDF) [file pone.0330172.s001.pdf]

Survey Information

(This survey is conducted only for research purpose)

Respondent's name: _ _ _ _ _ _ _ _ _ _   Respondent's ID: _ _ _ _ _ _   Date of collecting data: _ _ _ _ _ _

Socio-demographic Information

1. Sex: a. Male b. Female
2. Age :
3. Resident/Area: a. Rural b. Urban
4. What is the highest level of education you have completed?
   1. No formal schooling
   2. Less than primary school
   3. Primary school completed
   4. Secondary school completed
   5. High school completed
   6. College/University completed
   7. Post graduate degree
5. What is your marital status?
   1. Never married 2. Currently married
   3. Separated 4. Divorced
   5. Widowed
6. Which was you doing at past?
   1. Government employee
   2. Non-government employee
   3. Self-employed
   4. Non-paid
   5. No employment
7. Taking the past year, average monthly income of the household………………taka
8. How many people live in your household? ....

Physical Measurement

1. Height (cm):

2. Weight (kg):

Simplified Nutritional Appetite Questionnaire (SNAQ)

1. My appetite is:
   a) Very poor
   b) Poor
   c) Average
   d) Good
   e) Very good
2. When I eat
   a) I feel full after eating only a few mouthfuls
   b) I feel full after eating about a third of a meal
   c) I feel full after eating over half a meal
   d) I feel full after eating most of the meal
   e) I hardly ever feel full
3. Food tastes
   a) Very bad
   b) Bad
   c) Average
   d) Good
   e) Very good
4. Normally I eat
   a) Less than one meal a day
   b) One meal a day
   c) Two meals a day
   d) Three meals a day
   e) More than three meals a day

Morbidity Characteristics

1. Have you any of these health-related problems?

a) Visual Impairment
b) Hearing Problem
c) Musculoskeletal Pain
d) Bedsore
e) Food Allergy
f) Sense of Thirst
g) Dental Problem
h) Diabetes Mellitus
i) CVD (Cardiovascular Disease)
j) Hypertension

Health seeking behavior

1. How often do you visit a healthcare professional or seek medical advice?

a) Doesn't seek care
b) Several times per month
c) Once per month
d) Several times per year
e) Once per year
f) Less regular than once per year

Mini Nutritional Assessment

1. Has food intake declined over the past 3 months due to loss of appetite, digestive problems, chewing or swallowing difficulties?
   0) Severe decrease in food intake
   1) Moderate decrease in food intake
   2) No decrease in food intake

2. Weight loss during the last 3 months…
   0) Weight loss greater than 3 kg (6.6 lbs)
   1) Does not know
   2) Weight loss between 1 and 3 kg (2.2 and 6.6 lbs)
   3) No weight loss

3. Mobility
   0) Bed or chair bound
   1) Able to get out of bed / chair but does not go out
   2) Goes out

4. Has suffered psychological stress or acute disease in the past 3 months? 0) yes 2) no

5. Neuropsychological problems
   0) Severe dementia or depression
   1) Mild dementia
   2) No psychological problems

6. (I) Body Mass Index (BMI) (weight in kg) / (height in m)^2
   0) BMI less than 19
   1) BMI 19 to less than 21
   2) BMI 21 to less than 23
   3) BMI 23 or greater

(If BMI is not available, replace question 27.1 with question 27.2. Does not answer question 27.2 if question 27.1 is already completed)

7. (II) Calf circumference (CC) in cm
   0) CC less than 31
   3) CC 31 or greate

Physical Activity for Elderly (PASE)

Leisure time activity

1. Over the past 7 days, how often did you participate in sitting activities such as reading,watching TV or doing handcrafts?
[0.] NEVER ----- GO TO Q.#2 [1.] SELDOM--- (1-2 DAYS)
[2.] SOMETIMES---- (3-4 DAYS) [3.] OFTEN--- (5-7 DAYS)
1b. On average, how many hours per day did you engage in these sitting activities?
[1] <1 HOUR [2] 1-2 HOURS [3] 2-4 HOURS [4] >4 HOURS

2. Over the past 7 days, how often did you take a walk outside your home or yard for anyreason? For example, for fun or exercise, walking to work, walking the dog, etc.?
[0.] NEVER ----- GO TO Q.#3 [1.] SELDOM--- (1-2 DAYS)
[2.] SOMETIMES---- (3-4 DAYS) [3.] OFTEN---- (5-7 DAYS)
2a. On average, how many hours per day did you spend walking?
[1] <1 HOUR [2] 1-2 HOURS [3] 2-4 HOURS [4] >4 HOURS

3. Over the past 7 days, how often did you engage in light sport or recreational activities such as bowling, golf with a cart, fishing from a boat or pier or other similar activities?
[0.] NEVER ----- GO TO Q.#4 [1.] SELDOM--- (1-2 DAYS)
[2.] SOMETIMES---- (3-4 DAYS) [3.] OFTEN---- (5-7 DAYS)
3b. On average, how many hours per day did you engage in these light sport or recreational activities?
[1] <1 HOUR [2] 1-2 HOURS [3] 2-4 HOURS [4] >4 HOURS

4. Over the past 7 days, how often did you engage in moderate sport and recreational activities such as doubles tennis, ballroom dancing, hunting, ice skating, golf without a cart, softball or other similar activities?
[0.] NEVER ----- GO TO Q.#5 [1.] SELDOM---(1-2 DAYS)
[2.] SOMETIMES---- (3-4 DAYS) [3.] OFTEN---- (5-7 DAYS)
4b. On average, how many hours per day did you engage in these moderate sport and recreational activities?
[1] <1 HOUR [2] 1-2 HOURS [3] 2-4 HOURS [4] >4 HOURS

5. Over the past 7 days, how often did you engage in strenuous sport and recreational activities such as jogging, swimming, cycling, singles tennis, aerobic dance, skiing (downhill or cross-country) or other similar activities?

[0.] NEVER ----- GO TO Q.#6 [1.] SELDOM--- (1-2 DAYS)
[2.] SOMETIMES---- (3-4 DAYS) [3.] OFTEN--- (5-7 DAYS)

5b. On average, how many hours per day did you engage in these strenuous sport and recreational activities?

[1] <1 HOUR [2] 1-2 HOURS [3] 2-4 HOURS [4] >4 HOURS

6. Over the past 7 days, how often did you do any exercises specifically to increase muscle strength and endurance, such as lifting weights or pushups, etc.?

[0.] NEVER ----- GO TO Q.#7 [1.] SELDOM--- (1-2 DAYS)
[2.] SOMETIMES---- (3-4 DAYS) [3.] OFTEN--- (5-7 DAYS)

6b. On average, how many hours per day did you engage in exercises to increase muscle strength and endurance?

[1] <1 HOUR [2] 1-2 HOURS [3] 2-4 HOURS [4] >4 HOURS

Household activity

7. During the past 7 days, have you done any light housework, such as dusting or washing dishes?

[1.] NO [2.] YES

8. During the past 7 days, have you done any heavy housework or chores, such as vacuuming, scrubbing floors, washing windows, or carrying wood?

[1.] NO [2.] YES

9. During the past 7 days, did you engage in any of the following activities?

Work-related activity

10. During the past 7 days, did you work for pay or as a volunteer? [1.] NO [2.] YES

10a.How many hours per week did you work for pay and/or as a volunteer?................ HOURS

10b.Which of the following categories best describes the amount of physical activity required on your job and/or volunteer work?

[1] Mainly sitting with slight arm movements. [Examples: office worker, watchmaker, bus driver, etc.]

[2] Sitting or standing with some walking. [Examples: cashier, general office worker, light tool and machinery worker.]

[3] Walking, with some handling of materials less than 50 pounds. [Examples: construction worker]

[4] Walking and heavy manual work required materials weighing over 50 pounds. [Examples: stone mason, farm or general laborer.]

Diet Quality Questionnaire (DQQ)

DQQ	**Yesterday, did you eat any of the following foods:**		
**1**	Rice, Paratha or ruti?	**Yes**	**No**
**2**	Roti, corn or popcorn?	**Yes**	**No**
**3**	Potato, plantain, arum or sweet potato?	**Yes**	**No**
**4**	Dhal, chickpeas or khichuri?	**Yes**	**No**
	**Yesterday, did you eat any of the following vegetables:**		
**5**	Carrots or pumpkin?	**Yes**	**No**
6.1	Lal shak, pui shak, amaranth, spinach, or any other shak?		
**7.1**	Eggplant, lady finger, cauliflower, cabbage, long beans, green beans, or tomatoes?	**Yes**	**No**
**7.2**	Bottle gourd, pointed gourd, bitter gourd, bitter melon, or ash gourd?	**Yes**	**No**
**7.3**	White radish, kohlrabi, taro shoots, or green papaya?	**Yes**	**No**
	**Yesterday, did you eat any of the following fruits:**		
**8**	Ripe mango, ripe papaya, or orange musk melon?	**Yes**	**No**
**9**	Orange, malta, or pomelo?	**Yes**	**No**
**10.1**	Guava, pineapple, ripe banana, watermelon, jackfruit, custard apple, or apple?		
**10.2**	Jamrul, star fruit, koromcha, jujube, Java plum, litchi, or amra?		
	**Yesterday, did you eat any of the following sweets:**		
**11**	Sweet biscuits, cakes, misti pitha, halwa, or jilapi?	**Yes**	**No**
**12**	Mishti, chocolate, or ice cream?	**Yes**	**No**
	**Yesterday, did you eat any of the following foods of animal origin:**		
**13**	Eggs?	**Yes**	**No**
**14**	Paneer or cheese?	**Yes**	**No**
**15**	Yogurt or lassi?	**Yes**	**No**
**16**	Sausages?	**Yes**	**No**
**17**	Beef or goat meat?		
**18**	N/A		
**19**	Chicken, chicken liver, pigeon, duck, or quail?	**Yes**	**No**
**20**	Fish or dried fish?	**Yes**	**No**
	**Yesterday, did you eat any of the following other foods:**		
**21**	Peanuts or jackfruit seeds?	**Yes**	**No**
**22**	Chips or chanachur?	**Yes**	**No**
**23**	Instant noodles such as Maggi noodles or Pran's Mr. Noodles?		
**24**	Puri, singara, samucha, pakora, piaju, beguni, fried chicken, or chop?	**Yes**	**No**
	**Yesterday, did you have any of the following beverages:**		
**25**	Milk?	**Yes**	**No**
**26**	"Tea with sugar, coffee with sugar, chocolate milk, Milo, Horlicks, Complan or Ovaltine?"	**Yes**	**No**
**27**	Fruit juice, packet juice such as frooto or tang or shorbot?	**Yes**	**No**
**28**	Soft drinks such as Pepsi, Mojo, Sprite, or Fanta, or energy drinks such as Tiger?	**Yes**	**No**
	**Yesterday, did you get food from any place like...**		
**29**	KFC, CP, Pizza Hut, Helvetia, Burger King, Herfy, or other places that serve pizza or burgers?	**Yes**	**No**
